# Salmonella blood stream infections in a tertiary care setting in Ghana

**DOI:** 10.1186/s12879-014-0697-7

**Published:** 2014-12-21

**Authors:** Appiah-Korang Labi, Noah Obeng-Nkrumah, Naa Okaikor Addison, Eric Sampene Donkor

**Affiliations:** Department of Microbiology, Korle-Bu Teaching Hospital, Accra, West Africa, Ghana; Microbiology Department, University of Ghana Medical School, Accra, West Africa, Ghana

**Keywords:** Ghana, Salmonella, Antibiotic, Resistance, Risk factors

## Abstract

**Background:**

Despite the clinical significance of *Salmonella* infections, surveillance data worldwide remains limited and is more so exemplified by the lack of reports from Africa especially in eastern, central and western Africa. This study reports on Salmonella serotypes as significant causes of blood stream infections (BSI) and multidrug antibiotic resistance at Korle-Bu Teaching Hospital in Accra, Ghana.

**Methods:**

Antibiogram patterns, seasonal variations in disease incidence and predisposing factors for infection with Salmonella serotypes were analyzed retrospectively over a 4-year period from January 2010 to December 2013. Blood cultures were processed with BACTEC 9240 blood culture system. Speciation was done with BBL Crystal Enteric/Nonfermenter identification system®, and with slide agglutination using specific antisera. Antimicrobial susceptibility testing was carried out by the Kirby-Bauer disc diffusion method according to Clinical and Laboratory Standard Institute guidelines.

**Results:**

We report a 6.5% (n = 181/2768) prevalence of Salmonella bacteraemia at the Korle-Bu Teaching Hospital; with a preponderance of non-typhoidal salmonellae (NTS) over typhoidal salmonella (TS) (n = 115/181, 63.5% versus n = 66/181, 36.5%; P-value <0.002). Children under 5 years bore the brunt of the disease (n = 93/174, 53.4%). Resistance to ciprofloxacin (n = 1/127, 0.7%), amikacin (n = 3/81, 3.7%), and cefotaxime (n = 6/99, 6.1%) remained low, despite high levels of multidrug resistant Salmonella phenotypes (n = 81/181, 44.2%). In multivariate analysis, and among patients with Salmonella BSI, those < 1 year old had reduced risk of non-typhoidal infections [Odds ratio, 0.51; 95% confidence interval (95% CI), 0.16-0.92, P-value 0.021]. Similarly, patients with cefuroxime resistant strans were at increased risk of having multidrug resistant Salmonella BSI (OR, 8.97; 95% CI, 3.62-24.15; P-value, 0.001).

**Conclusions:**

Salmonellae, predominantly NTS, account for a reasonable low proportion of positive blood cultures in our tertiary care setting; but with significant multidrug resistant phenotypes and low ciprofloxacin and cefotaxime resistance.

**Electronic supplementary material:**

The online version of this article (doi:10.1186/s12879-014-0697-7) contains supplementary material, which is available to authorized users.

## Background

Invasive salmonellosis remains a global public health problem. Global estimates in 2000 showed that typhoid fever accounted for 21, 000 000 illnesses and 216, 000 deaths, whilst 5, 412 744 illnesses were attributable to paratyphoid fever [1]. The epidemiology of invasive salmonellosis varies worldwide. In Europe, non-typhoidal Salmonella (NTS) mainly *Salmonella* Enteritidis predominate but are rarely associated with systemic disease other than diarrhoea except for severely immunocompromised patients) [2]. In South East Asia, the predominant organism is *Salmonella* Typhi [1],[3], whilst in Africa NTS predominates [1],[4]-[8]. The disease is common in developing countries and concomitant with poor public health and low socio economic indices [9],[3]. Thus the bulk of the disease burden is seen in South East Asia, Africa and Latin America [3]. Its’ epidemiology is also affected by seasonal variations [2],[10]. In India peak incidence of *Salmonella* Typhi occurs between April-June (dry season) followed by July-September (monsoon season). In Africa the epidemiology of invasive salmonellosis has been linked with malaria infections [4],[8],[11],[12].

In Ghana, there is paucity of epidemiological data on invasive salmonellosis with a few studies suggesting high disease burden in children. A recent report found invasive non-typhoidal salmonellosis in 10% of hospitalized children [5], whilst another study documented a prevalence of 57% in infant bactereamia [4].

*Salmonella* species are increasingly evolving antimicrobial resistance to several commonly used antimicrobial agents. This phenomenon which started with a report of chloramphenicol resistant *Salmonella* Typhi from India in 1972 [13] has increased to the extent that multi-drug resistant strains are now circulating globally [14]. This is the result of indiscriminate use of antibiotics in areas of high transmission or endemicity [14]. The problem of MDR Salmonella increases the challenge in the management of the disease in endemic regions by increasing morbidity and mortality as well as cost of treatment.

Surveillance data worldwide remains limited (2), and is more so exemplified by the lack of data from Africa especially in eastern, central and western Africa (1). Such data is relevant in decision making by public health officials for disease prevention and control programmes (1). In Ghana the situation is no different. In this study, we have documented the epidemiology of invasive salmonellosis in a tertiary hospital setting in Ghana. Our primary outcomes were to report the prevalence, circulating serotypes, antimicrobial resistance patterns, seasonal variations in disease incidence and some predisposing factors for this infection.

## Methods

### Study setting

The retrospective study was conducted in Korle-Bu Teaching Hospital (KBTH), a 2000-bed tertiary teaching hospital with about 200 admissions per day [15]. The hospital covers all medical specialties and provides referral healthcare services to an estimated population of 24 million Ghanaians. The central outpatient department records about 29,757 patient turnout per month [15]. The bacteriology unit of the Microbiology Department of KBTH processes over 40,000 clinical cultures annually.

### Study design

From January 2010 through December 2013, we studied all clinical bloodstream isolates of salmonellae collected at the bacteriology unit of the Central Microbiology Laboratory in KBTH. Two sampling approaches were used. First, all Salmonella isolates recovered from blood cultures of patients visiting the microbiology laboratory of KBTH were characterized and analysed. Second, we reviewed laboratory records of all patients with Salmonella BSI for microbiological data. *Salmonella* species were selected based on the following criteria: (i) confirmed as the causative agent of the infection for which blood cultures were performed, and (ii) identified as first isolate per patient within study period. Multiple isolates per patient were considered only if antibiogram was different than previous, and their bacteraemia episodes were more than 3 months separate.

### Patients’ record review

To provide accurate information, patients and isolates data were abstracted in the following two steps, (i) manual work through of laboratory records, and (ii) physician-assisted medical reconciliation of data. Data were retrospectively reviewed and compared. When bacteraemic episodes with Salmonella were identified, we categorized those with typhoid and non-typhoid isolates. Data were retrospectively reviewed and compared. Subsequently, univariate and multivariate analysis were conducted to compare patients with Salmonella BSI caused by typhoidal strains to their counterparts with non-typhodal isolates using patients’ data from laboratory records as independent predictor variables. Similar analysis were also conducted to compare a second case group comprising patients with BSI caused by MDR Salmonella to those with non-MDR Salmonella BSI. Each patient was included only once for each outcome. Patients’ groups were compared regarding the following: demographics (age, gender), sickle cell disease, month and year of infection as well as patients’ hospital and assigned department of care. We also collected data on variables related to infections: types of *Salmonella* species and antibiogram.

### Specimen, culture and identification

During the study period, and as part of hospital routing care, 23,708 patients submitted blood cultures for bacteriological investigations. Speceimens were processed with BACTEC 9240 blood culture system (Becton Dickinson, NJ, USA) according to manufacturer’s instructions. A total of 2,768 blood cultures were positive for various infections. Subcultures were made on blood, chocolate and MacConkey agar; and were incubated aerobically at 37°C for 20 hours. Typical lactose non-fermenting colonies were speciated with standard bacteriologic reactions and BBL Crystal Enteric/Nonfermenter identification system® (Becton Dickinson, NJ, USA). Salmonellae were confirmed with slide agglutination using specific antisera (Himedia Laboratories, India).

### Susceptibility testing

The susceptibilities of isolates to the antimicrobial agents ampicillin (10 μg), cefuroxime (30 μg), cefotaxime (30 μg), chloramphenicol, (30 μg), cotrimoxazole (25 μg), gentamicin (30 μg), amikacin (30 μg), ciprofloxacin (5 μg), and levofloxacin (5 μg) (HiMedia Laboratories, India) were determined with Kirby-Bauer disc diffusion method in accordance with the Clinical and Laboratory Standards Institute (CLSI) guidelines [16]. The reference strain *E. coli* ATCC 25922 was included as quality control in the susceptibility assays. According to the international standard definitions for acquired resistance, and relative to the panel of antibiotics tested, multidrug resistant (MDR) phenotype was defined as *in vitro* non-susceptibility to ≥1 agent in ≥3 antimicrobial categories [17]: penicillins, cephalosporins, beta-lactamase inhibitor combinations, fluoroquinolones, aminoglycosides, chloramphenicol, folate pathway inhibitors, tetracyclines, macrolides and glycopeptides.

### Statistics

Data from laboratory investigations were captured into Microsoft ACCESS to generate a database, and exported into Statistical Package for Social Sciences (SPSS, Version 20.0) for data editing and statistical analyses. Continuous data were compared using student’s t-test, analyses of variance (ANOVA) or Mann–Whitney U test, (respectively for normalized and non-parametric distributions), with point estimates of statistical significance indicated by with 2 tailed P-values <0.05. Categorical data were compared across study parameters using Chi-square with Maracuilo’s post hoc tests for multiple comparisons, or the Fisher’s exact test. Correlations were assessed with Pearson coefficient (r) or Spearman’s rho (r_s_) or their coefficient of determination (r^2^ or r_s_^2^) where appropriate. Univariate comparisons were computed with Chi-square tests and unadjusted Odds ratios (OR) at 95% confidence interval (CI). From univariate analyses, variable with a P-value <0.05 were analysed in a multivariate logistic regression models to identify independent risk factors. Predictive accuracy of the models was evaluated by Hosmer and Lemeshow goodness-of-fit test with P-value >0.05 suggesting that the model predicts accurately on average. The area under the ROC (Receiver Operating Characteristic) Curve > 0.7 was used to analyse the discriminatory capability of bactereamia with typhoidal salmonella or MDR salmonellae versus their respective controls.

### Ethical considerations

Ethical approval was not required as the study was regarded as part of routine surveillance measures for infection control by the Ethical and Protocol Review Committee of University of Ghana Medical School, College of Health Sciences. Considering the retrospective nature of the study, we could not obtain patients consent for use of clinical data. Nevertheless, on receipt of isolates and patients’ data, we de-identified all patients to ensure anonymity. We also allotted arbitrary numbers to all isolates assigned to the study.

## Results

### Pravalence of salmonellae

During the study period, 23,708 blood cultures were submitted for bacteriological investigations. A total of 2,768 blood cultures were positive for various infections. From these, 181 (6.5%) non-duplicate salmonellae were recovered in 2010 (n = 37/749, 4.9%), 2011 (n = 53/701, 7.5%), 2012 (n = 63/796, 7.9%), and 2013 (n = 28/432, 6.5%). Thus 181 patients were included in this study (98 males and 83 females), with a mean age of 9.5 years (interquartile range, 6 years). The prevalence of TS and NTS were respectively 2.4% [n = 66/2768; 95% confidence interval (CI), 1.93-3.13] and 4.10% (n = 115/2768; 95% CI, 3.10-4.51); with the total number of TS being significantly lower than that of NTS (n = 66/181, 36.5% versus 115/181, 63.5%; P-value <0.002). Overall, we did not observe correlation between salmonellae prevalence and study years [r_s_(2) = 0.4, P-value = 0.617]. Table 1 shows the distribution of *Salmonella* species across study years. *Salmonella* Enteritidis (n = 93/181, 51.1%), and *S.* Typhi (n = 48/181, 26.5%) were the predominant serotype. Others were *S.* Typhumurium (n = 22/181, 11.5%) and *S.* Paratyphi (n = 18/181, 9.9%). Marascuilo’s post hoc test was conducted to compare the proportions of salmonellae recovered across the 4 study years. Over the study period the yearly populations of each salmonella serotype had similar proportion of defects except for *S.* Enteritidis for which more isolates were recovered in 2013 compared to 2010 (P-value = 0.018), and in 2011 compared to 2013 (P-value = 0.019).

**Table 1:** **A four- year distribution of salmonellae recovered from blood cultures submitted to microbiology laboratory of Korle-Bu Teaching Hospital**

Isolates	Number of ***Salmonella*** species ^a^ (%)										
	Total (n = 181)	Crx-res.	2010		2011		2012		2013		P-value ^b^
			Total (n = 37)	Crx res.	Total (n = 53)	Crx res.	Total (n = 63)	Crx res.	Total (n = 28)	Crx res.	
Typhoidal Salmonella	66 (36.5)_a_	11 (16.7)_a_	14 (37.8)	4 (28.5)	12 (22.4)	4 (30.7)	27 (44.8)	2 (7.4)	13 (22.4)	1 (7.6)	0.1322
*S.* Typhi	48 (26.54)	7 (14.5)	8 (21.6)	2 (25.0)	7 (14.8)	2 (25.0)	25 (61.9)	2 (3.3)	8 (28.5)	1 (12.5)	0.8056
*S.* Paratyphi *A*	12 (6.6)	4 (33.3)	3 (8.1)	2 (66.6)	3 (5.5)	2 (66.7)	1 (1.6)	0	5 (17.8)	0	0.0441
*S.* Paratyphi *B*	6 (3.3)	0	3 (8.1)	0	2 (3.7)	0	1 (1.6)	0	0	0	0.1988
Non-typhoidal Salmonella	115 (63.5)_b_	21 (18.2)_a_	23 (62.1)	5 (21.7)	41 (77.3)	11 (23.4)	36 (57.1)	4 (11.1)	15 (53.5)	1 (6.7)	0.1322
*S.* Enteritidis	93 (51.2)	18 (19.3)	16 (25.2)	4 (25.0)	33 (61.1)	9 (27.2)	29 (46.0)	4 (13.8)	15 (53.5)	1 (6.7)	0.129^c^ 2010*2013, P = 0.018; 2011*2013, P = 0.019
*S.* Typhumurium	22 (12.2)	3 (13.6)	7 (18.9)	1 (14.2)	8 (14.0)	2 (25.0)	7 (6.1)	0	0	0	-

_a_Crx res, cefuroxime resistant; _b_P-value determined with Marascuilo’s comparisons post hoc tests for multiple proportions; ^c^ *P,* P-value for significant pairwise comparisons between study years;

*Differing subscripts within a column indicate significant difference at P-value < 0.05; SpearmansRanked Correlation between prevalence of Salmonella isolates and study years, r_s_(2) = +0.4; P-value = 0.617.

### Cefuroxime resistant phenotypes

As a crude measure to determine beta-lactamase producing phenotypes, we examined the resistance pattern of the salmonellae to cefuroxime (30 μg) (Table 1). The 4-year prevalence of cefuroxime resistance was similar among TS and NTS (n = 11/66, 16.7% versus n = 21/115, 18.2% respectively; P-value = 0.786). Marascuilo’s comparisons showed homogeneity in the proportion of cefuroxime resistant strains across the four yearly populations of typhoidal salmonellae (P-value = 0.1115). Conversely, although we recorded significant difference (P-value = 0.0161) in the proportion of cefuroxime resistance for the yearly populations of non-typhoidal salmonellae, no difference between any two particular years was significant (marascuilo’s post hoc > 0.05 for all pairwise comparisons). For TS and NTS, the Spearman’s ranked test showed no correlations between cefuroxime prevalence and study years [TS, r_s_(2) = −0.6, P-value = 0.438; NTS, r_s_(2) = −0.8; P-value = 0.231].

### Seasonal variations in prevalence of salmonellae

**Figure 1:**
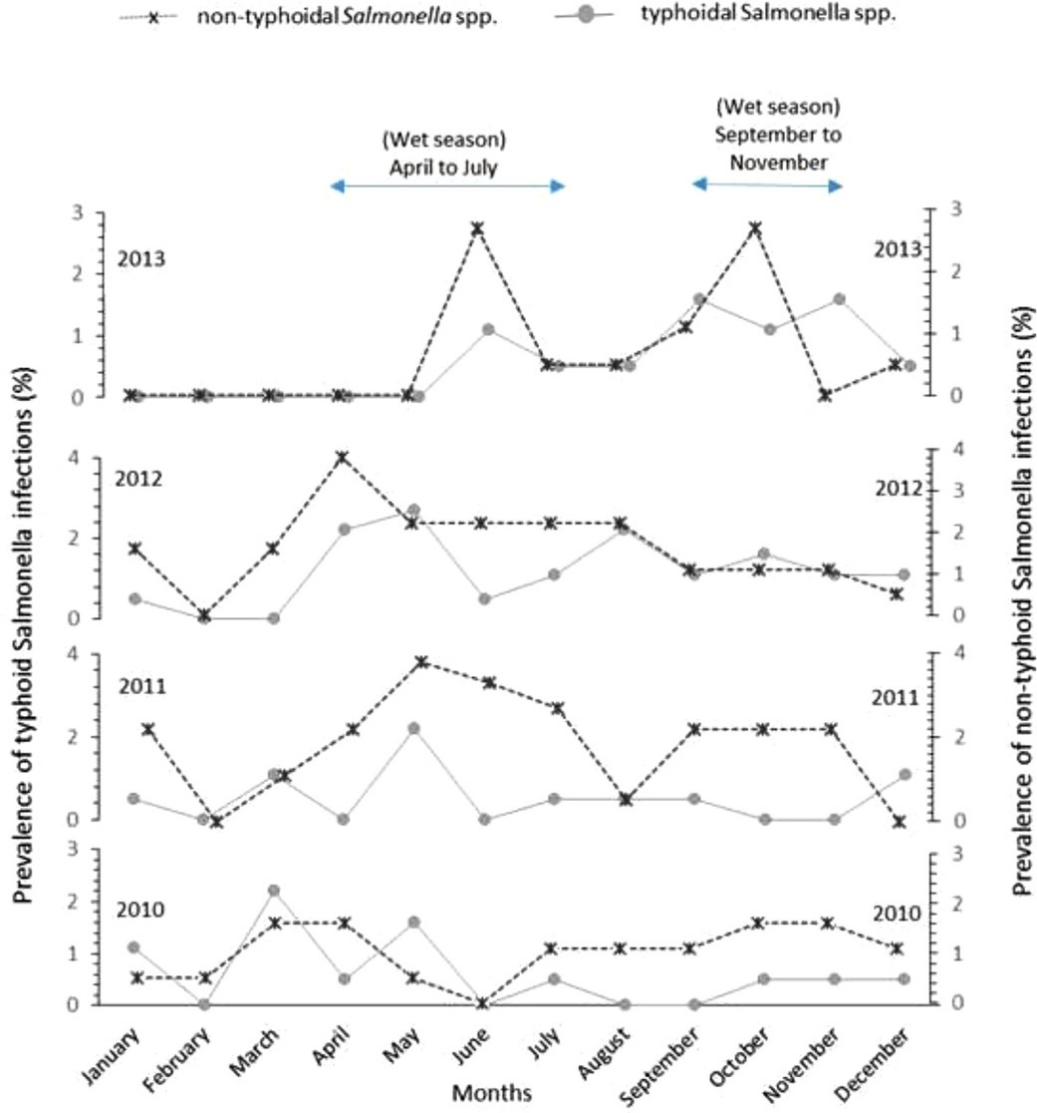
**Seasonal shifts in bactereamia infections caused by typhoidal (TS) and non-typhoidal Salmonella (NTS) from 2010 through 2013.** Monthly variations (X-axis) in Salmonella infections for each year are reported as percentage prevalence (Y-axis); prevalence for TS and NTS infections are respectively shown with dotted and plain lines. We observed a seasonal trend for TS, but not NTS, with rainfall pattern. The peak seasons for TS bactereamia were observed in March though July and from September through November; and coincided with the two rainy seasons in Ghana — April to July, and September to November.

Figure 1 shows the monthly distribution of TS and NTS over a four-year study period. There was seasonality for TS, but not NTS, with rainfall pattern. The peak seasons for TS bactereamia were observed in March though July and from September through November; and coincided with the two rainy seasons in Ghana — April to July, and September to November (Figure 1). For instance, the highest TS prevalence peaks were observed in March (2.2%) and May (2.0%) for 2010; and in May (2.3%) for 2011. April (2.2%), May (2.75%) and August (2.2%) recorded the highest bactereamic episodes in 2012; whereas Sepetember (1.6%) and November (1.6%) accounted for the highest prevalence in 2013. Altogether, non-typhoidal salmonellae (NTS) appeared to be more fairly distributed across the months and over the years. For the particular years under study, we sought to examine the correlation between months and the number of salmonellae isolated. The number of TS recovered in 2013, unllike the other years, showed some significant degree of covariation with months; and the direction of covariation was positive. More so, 51.9% of the variance in the number of TS was coupled with advancing months (r_s_ = 0.799; r_s_^2^ = 0.519; P-value = 0.001). On the contrary, no significant trend in correlation was observed between the number of non-typhoidal Salmonella (NTS) and months of isolation for any particular year.

### Antibiotic susceptibility results

The *in vitro* susceptibility results of salmonellae to potentially useful antimicrobial agents are shown in Table 2. Overall, ampicillin, chloramphenicol and tetracycline were the least effective antibiotics with over 70% of TS and NTS resistant to each antimicrobial. For TS, no resistance was found to cefotaxime and the ciprofloxacin; whereas gentamicin, amoxicillin/clavulanate and amikacin were respectively ineffective against 4.1% (n = 1/24), 8.3% (n = 1/12) and 9.1% (n = 1/11) of the isolates. Similarly, NTS were mostly susceptible to the ciprofloxacin (n = 134/135, 99.2%), amikacin (n = 70/71, 98.6%), cefotaxime (n = 93/99, 93.9%), and gentamicin (n = 84/93, 90.3%). The percentage resistance between TS and NTS were similar for all tested antibiotics except cotrimoxazole — with a smaller percentage of TS than NTS being resistant to this antimicrobial (TS, 15.5%, n = 27/174 versus NTS, 62.1%, n = 54/87; P-value = 0.002). Chloramphenicol was the only antibiotic to which salmonellae resistance appears to be increasing; we noted a positive correlation between time over 4 years and percentage of NTS resistant to this antimicrobial (r_s_ = +1, P-value = 0.0001).

**Table 2:** **Antibiotic susceptibility patterns of typhoidal and non-typhoidal salmonellea recovered from blood cultures**

Antibiotics	Proportion of resistant ***Salmonella*** species (%)												
	Total		P-value	2010		2011		2012		2013		Correlation coefficient ^c,d^	
	TS ^a^	NTS ^b^		TS	NTS	TS	NTS	TS	NTS	TS	NTS	TS	NTS
Ampicillin	27/43 (62.7)	81/103 (78.6)	0.0466	12/14 (85.7)	20/23 (83.3)	9/13 (69.2)	28/37 (75.6)	3/13 (23.1)	23/30 (76.6)	3/10 (30.0)	10/13 (76.9)	r_s_ = −0.8, P = 0.2	r_s_ = −0.2, P = 0.8
cefuroxime	11/63 (17.4)	21/104 (20.2)	0.6636	4/14 (28.5)	5/20 (25.0))	4/13 (30.7)	11/38 (28.9)	2/26 (76.9)	4/31 (12.9)	1/10 (10.0)	1/15 (66.7)	r_s_ = −0.2, P = 0.8	r_s_ 0.4, P = 0.6
Cefotaxime	0	6/99 (6.1)	-	0	1/23 (4.5)	0	3/41 (7.3)	0	2/35 (57.1)	0	0	-	r_s_ = −0.2, P = 0.8
Amoxicillin/ Clavulante	1/12 (8.3)	11/20 (55.5)	<0.01	NT	2/5 (40.0)	NT	6/7 (85.7)	1/12 (8.3)	3/8 (37.5)	0	0	-	r_s_ = −0.8, P = 0.2
Gentamicin	1/24 (4.1)	9/91 (9.9)	0.3892	0	2/23 (9.1)	0	5/35 (14.3)	1/24 (4..2)	2/33 (6.6)	0	0	r_s_ = 0.26, P = 0.74	r_s_ = −0.8, P = 0.2
Amikacin	1/11 (9.1)	2/71 (2.8)	0.3025	0	0	0	1/38 (2.6)	0	1/33 (3.3)	1/11 (9.1)	0	r_s_ = 0.77, P = 0.23	r_s_ =0.26, P = 0.75
Chloramphenicol	27/44 (61.4)	66/90 (73.3)	0.1579	10/13 (76.9)	10/19 (52.6)	12/13 (92.3)	25/33 (75.7)	5/18 (27.7)	22/27 (81.5)	NT	9/11 (81.8)	-	r_s_ =1 P = 0.001
Cotrimoxazole	24/174 (13.8)	54/87 (65.5)	0.0002	8/145 (5.5)	10/18 (55.5)	7/13 (53.8)	19/36 (52.7)	8/10 (80.0)	17/21 (80.9)	1/6 (16.7)	8/12 (66.7)	r_s_ =0.4, P = 0.6	r_s_ = 0.6, P = 0.4
Tetracycline	26/37 (70.3)	60/83 (72.3)	0.8204	9/14 (64.2)	14/17 (82.3)	9/13 (69.2)	22/32 (68.8)	8/10 (80.0)	16/23 (69.5)	0	8/11 (72.7)	r_s_ = −0.2, P = 0.8	r_s_ = −0.2, P = 0.8
Ciprofloxacin	0	1/ 135 (0.7)	-	0	0	0	1/135 (0.7)	0	0	0	0	-	-

^a^TS, typhoidal salmonellae; ^b^NTS, non-typhoidal salmonellae; ^c^r_s,_ Spearman’s Correlaltion Coefficient, measures the overall significant increase or decrease in antibiotic resistance level over the 4-year period; ^d^P, P-values at <0.05 are significant.

The number of salmonellae tested for susceptibility differ for each antibiotic. Results are thus recorded as proportions (number of resistant strains per total isolates tested for a particular antibiotic) with percentages in parenthesis.

### Multidrug resistance salmonellae

Eighty-one (44.8%) salmonellae expressed multidrug resistant phenotypes. The multidrug resistant strains (MDRs) were significantly more in NTS (n = 59/115, 51.3%) compared to TS (n = 22/66, 33.3%); with cefuroxime resistant phenotypes more abundant in TS (n = 9/22, 40.9%) than in NTS (n = 18/59, 30.5%). Some MDRs (TS, n = 4/22, 18.2%; NTS, n = 11/59, 18.6%) remained resistant to all except one class of the antimicrobials tested. These were susceptible to gentamicin (TS, n = 3/4, 75%; NTS, n = 9/11, 81.8%), amikacin (TS, n = 4/4, 100%; NTS, n = 11/11, 100%), the ciprofloxacin (TS, n = 4/4, 100%; NTS, n = 6/6, 100%), and cefotaxime (TS, n = 4/4, 100%; n = 6/11, 54.5%).

### Age distribution of salmonellae strains

**Figure 2:**
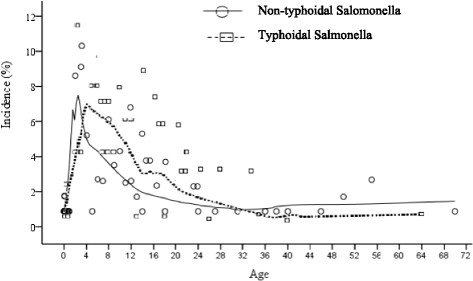
**Incidence of Salmonella bactereamia per age distribution.** Generated with Locally Weighted Scatterplot Smoothing (LOESS) Tricube kernel smooth fit line with 50% of points to fit. The results show similarities in the incidence of TS and NTS with age. Overall, TS and NTS increased with age and were highest in children below 5 years, but declined gradually over ages 5 through 32, remaining fairly constant among the over 33 to 72-year-olds.

In Figure 2, we examined the age-specific incidence of TS and NTS across study patients using locally weighted scatter plot smoothing (LOWESS) fit lines. Two observations are noteworthy. First, the results show similarities in the incidence of TS and NTS with age. Second, the occurrence of Salmonella bacteraemia show contrasting patterns for patients at extremes of ages. Specifically, TS and NTS bactereamias increased with age and were highest in children below 5 years, but declined gradually over ages 5 through 32, remaining fairly constant among the over 33 to 72-year-olds. A Pearson Product–moment Correlation was run to determine the relationship between incidence of Salmonella bactereamia and age. Whereas a negative correlation was observed between incidence of TS and age, only 21.5% of the incidence variance was accounted for by increases in patients’ age (r = −0.464; r^2^ = 0.2153; P-value = 0.0297). In contrast, no demonstrable measure of covariation was recorded between NTS and age (r = −0.1795; r^2^ = 0.0322; P-value = 0.2613). Overall, the highest number of culture positive cases for TS and NTS was observed in children 1 year of age (TS, n = 7/66, 10.6%; NTS, n = 20/115, 17.4%), and the least from amongst adults >33 years.

### Predictors of Salmonella bactareamia

In Table 3, patients with typhoidal bactreamia were matched to those with non-typhoidal bactreamia to identify variables that increase the likelihood of developing TS BSI. Similar analysis were performed to determine factors associated with increased risk of Salmonella BSI by MDR salmonellae. Notable findings from univariate comparisons were that patients with typhoidal salmonellae were less likely to be children under 1 year old [Odds ratio (OR), 0.39; 95% confidence interval (CI), 0.18-0.84; P-value, 0.014]; and were less often infected with MDR strains (OR, 0.47; 95% CI, 0.25-0.89; P-value, 0.001). The MDR salmonellae were more frequently cefuroxime resistant (OR, 8.35; 95% CI, 3.02-23.05; P-value, 0.001). Patients with typhoidal bactereamia had been more often presumptively diagnosed with sepsis (OR, 1.92; 95% CI, 0.94-3.96; P-value, 0.072) or enteric fever (OR, 3.79; 95% CI, 1.18-12.09; P-value, 0.021). The MDR salmonellae were more often recovered from patients presumptively diagnosed with enteric fever (OR, 3.99; 95% CI, 1.05-15.05; P-value, 0.029). Non-MDRs were more frequently observed among patients suspected of sepsis (n = 39/64, 60.9% versus n = 23/67, 34.3%; P-vaue, 0.002). Table 4 shows risk factors from multivariate analysis for Salmonella BSI infection with typhoidal strains compared to non-typhoidal isolates and with MDRs compared to non-MDRs. In the end, among patients with Salmonella BSI, those < 1 year old had reduced risk of non-typhoidal infections [Odds ratio, 0.51; 95% confidence interval (95% CI), 0.16-0.92, P-value 0.021]. Similarly, patients with cefuroxime resistant strans were at increased risk of having MDR Salmonella BSI (OR, 8.97; 95% CI, 3.62-24.15; P-value, 0.001).

**Table 3:** **Baseline characteristics of patients**

Variables	Number	Salmonella bactereamia				Salmonella isolates in BSI ^b^			
		Typhoidal (66)	Non-typhoidal (115)	Odds ratio (95% CI ^a^)	P-value	MDRs (n = 81)	Non-MDRs (n = 100)	Odds ratio (95% CI)	P-value
Male gender	98	34	64	0.85 (0.46-1.55)	0.590	50	48	1.74 (0.96-1.17)	0.065
Age (±SD)^c^		10.3 ± 2.5	9.0 ± 3.79	1.02 (0.82-1.76)	0.051	9.1 ± 1.8	9.3 ± 2.1	0.93 (0.81-1.65)	0.295
Age^d^									
0-1	49	11	38	0.39 (0.18-0.84)	0.014	23	26	1.19 (0.61-2.31)	0.610
1-5	44	17	27	1.11 (0.55-2.25)	0.741	19	25	0.96 (0.48-1.92)	0.920
6-13	58	28	30	2.07 (1.08-3.96)	0.026	31	27	1.80 (0.95-3.41)	0.070
>13	23	8	15	0.90 (0.36-2.26)	0.823	7	16	0.51 (0.20-1.32)	0.162
Month of infection^e^									
Jan-Mar	27	10	17	1.02 (0.44-2.40)	1.0	21	6	5.48 (2.09-14.36)	0.001
April-June	62	21	41	0.84 (0.44-1.60)	0.603	31	31	1.38 (0.74-2.55)	0.305
July-sept	47	17	30	0.98 (0.49-1.96)	1.0	21	26	0.99 (0.51-1.94)	1.0
Oct-Dec	45	18	27	1.22 (0.61-2.44)	0.571	13	32	0.41 (0.19-0.84)	0.013
Year of infection									
2010	37	23	14	3.85 (1.82-8.20)	0.001	21	6	5.48 (209–14.36)	0.001
2011	54	13	41	0.44 (0.22-0.91)	0.02	31	23	2.07 (1.08-3.96)	0.025
2012	62	26	36	1.42 (0.75-2.68)	0.269	22	40	0.55 (0.29-1.05)	0.070
2013	28	13	15	1.63 (0.72-3.69)	0.233	7	21	0.35 (0.14-0.88)	0.02
Sickle cell disease^f^	27	6	21	0.45 (0.17-1.19)	0.104	16	11	1.51 (0.64-3.56)	0.342
Physicians’ diagnosis^f^									
Sepsis	62	27	35	1.92 (0.94-3.96)	0.072	23	39	0.33 (0.17-0.68)	0.002
Enteric fever	14	9	5	3.79 (1.18-12.09)	*0.022	11	3	3.99 (1.05-15.05)	0.029
PUO	20	5	15	0.55 (0.18-1.63)	0.281	7	13	0.46 (0.17-1.23)	0.116
BEMP	13	3	10	0.51 (0.12-1.95)	*0.378	5	8	0.56 (0.17-1.83)	0.33
Undiagnosed	71	21	50	0.56 (0.27-1.16)	0.118	35	36	0.85 (0.42-1.69)	0.646
Patient location^g^									
Child health	85	29	56	1.23 (0.56-2.71)	0.596	42	43	1.06 (0.52-2.22)	0.862
Medical	14	7	7	2.28 (0.75-7.00)	*0.225	4	10	0.39 (0.11-1.28)	0.108
NICU	2	0	2	-	-	0	2	-	-
Surgical	2	0	2	-	-	1	1	-	-
PML	25	5	20	0.44 (0.15-1.26)	0.122	13	12	1.17 (0.49-2.80)	0.729
KBTH patients^h^	102	36	66	0.76 (0.41-1.45)	0.416	47	55	0.59 (0.24-1.38)	0.223
Crx resistant salmonellae^i^	32	11	21	0.89 (0.40-1.99)	0.79	27	5	8.35 (3.02-23.05)	0.001
Typhoidal salmonella	66	-	-	-	-	22	44	0.47 (0.25-0.89)	0.02

^a^CI, confidence interval; ^b^BSI, blood stream infections; MDR, multidrg resistant strains; ^c^SD, standard deviation; ^d^Recorded for 64 TS and 110 NTS, and 75 MDR and 96 non-MDRs; ^e^Jan-Mar, January to March; Sep,september; Oct-Dec, October to December; ^f^Recorded for 47 TS and 85 NTS, and 67 MDRs and 64 non-MDRs; PUO, pyrexia of unknown origin; BEMP, bone infections, endocarditis, meningitis, pneumonia; ^g^Recorded for 42 TS and 87 NTS, and 63 MDRs and 66 non-MDRs; NICU, neonatal intensive care unit; PML, Princess-Marie Louis Children hospital; ^h^KBTH, Korle-Bu Teaching Hospital, recorded for 42 TS and 87 NTS, and 63 MDRs and 66 non-MDRs; ^i^Crx, cefuroxime, tested for 63 TS and 104 NTS, and 80 MDRs and 87 non-MDRs.

*P-vaue with Fisher’s exact test.

**Table 4:** **Risk factors for Salmonella bactreamia**

Risk factor	Level	Odds ratio (95% CI )	P-value
*TS versus NTS bactereamia*			
<1 years	Yes/No	0.51 (0.16-0.92)	0.021
*MDR Salmonella BSI versus non-MDRs*			
Crx resistant salmonellae	Yes/No	8.97 (3.62-24.15)	0.001

TS, typhoidal Salmonella; NTS,non-typhoidal salmonella; MDRs, multidrug resistant strains; Crx, cefuroxime; CI, confidence interval, BSI, blood stream infections.

## Discussion

When poor public health prevails in low socio-economic settings, it is natural to speculate that it will have an impact on the health status of the people. This may manifest itself as an increase in the incidence of diseases, including Salmonella infections. Over the last decade, several studies have demonstrated the clinical significance of salmonellae causing infections in hospitals. Despite this rise, there are very few reports from West Africa, and Ghana in particular, on the occurrence of Salmonella infections.

To our knowledge this is the first study conducted in Ghana across children and adult populations to document the prevalence, circulating serotypes, antimicrobial resistance patterns, and seasonal variations in disease incidence, as well as predisposing factors for blood stream infections with salmonellae. Our study reveals an overall low prevalence (6.5%) of Salmonella bactereamia among patients in KBTH from January 2010 through December 2013. Approximately 64% (n = 115/181) of the isolates were non-typhoidal Salmonella. Our finding of salmonellae in KBTH is lower compared with that documented elsewhere in Ghana (50%) [18], but higher than those reported in other salmonellae-affected institutions in India (0.69%) [10], and Tanzania (4.7%) [8]. The Korle-bu Teaching Hospital from which these isolates were recovered serves as a referral centre for primary and secondary health care facilities mostly from the Southern sector of Ghana. Thus most of these patients are likely to have been treated with prior antibiotics which is likely to reduce the culture yields.

There was a significant (P = 0.0002) prevalence of NTS (4.1%) compared to TS (2.4%) over the study period. The predominance of NTS over TS could be explained in part by the preponderance of children in our study population (86.8%, n = 151/174). In 2002, the estimated prevalence of TS (27.6%) predominated over that of NTS (40.7%) in patients with bacterial bloodstream infections in rural hospitals in Ghana [18]. The figures were lower (NTS, 18.8%; TS, 31.3%) 7 years later in the same study, but still higher for TS contrary to the results described in this study. In our study most patients were children, while patients who had their diagnostic samples collected in the previous rural study were primarily adults with a reported interquartile range of 26 years. Using comparable methodology, our finding is much closer to the 59% NTS and 25% TS previously reported among bacteraemia children in this institution [19]. In a separate Ghanaian study that included 99 villages and 1,456 hospitalized children <15 years of age, the prevalence of TS, driven largely by *S*. Typhi, was 2.4% [20]. We cannot directly compare our figures with those observed in that study because they did not assess NTS; nevertheless, they seem to provide a similar qualitative picture, in that the incidence of typhoidal fever is low in children. Elsewhere in Africa, NTS has been shown to be a prominent cause of blood stream infections in children [7],[21]-[23]. The interrelationship between severe malaria, NTS, and HIV infection has been described [24]. However, a much more consistent association has been suggested between bactereamia due to NTS and severe malaria [24],[25]. In this study, we are unable to determine the proportion of patients with HIV. But given that children in Ghana are prone to develop malaria due to the endemicity of the disease in this region, our finding is perhaps be more explained by the association of malaria with NTS.

Our study shows that TS and NTS bactereamia increased with age and were highest in children below 5 years, but declined gradually after the age of five years. This is corroborated by other studies in Ghana [20]. The high prevalence of Salmonella bloodstream infections in children under five could be explained by the relative poor hygiene observed by children within this group. We also observed that children under 1 year were less likely to be infected with TS and were less often infected with MDR strains; this is at variance with another study in Ghana which showed that the frequency of TS was low in children <2 years [20].

Peak isolation of Salmonella bloodstream infections occurred between the periods of March and November. This period marks the rainy season in Ghana [26], a time when floods occur leading to contamination of water bodies and an increase in the risk of developing Salmonella infections. Similar associations have been found in India [10] and Malawi [23] where the increase in incidence of invasive Salmonella infections has been associated with the rainy season.

Overall resistance of NTS and TS to ampicillin, tetracycline and chloramphenicol was over 70%. Such high resistance rates of Ghanaian salmonellae for ampicillin, chloramphenicol and tetracycline have been recently described by Marks et al. [27]. There are other reports in Ghana where high resistance of salmonellae to these agents have been documented [5],[18],[20]. High resistance of salmonellae to antimicrobial agents have also been documented elsewhere in subsaharan Africa [23],[28],[27]. In this current study, resistance of NTS and TS to ciprofloxacin and cefotaxime (recommended agents for treatment in Ghana) [29] was very low compared to reports from India where high resistance of about 13% to ciprofloxacin have been documented [30]. The low resistance ciprofloxacin and cefotaxime in Ghana have been coroborrated by others [18],[20],[31]. The relatively recent introduction of these agents as treatment options for invasive Salmonellosis may have contributed to such low resistance. Morover, resistance to these agents on the African continent is not widespread but is said to be an emerging problem [27].

There are some potential limitations of this study that should be discussed briefly. Blood stream infections from Korle-Bu were identified as including TS and NTS which constituted 6.5% of blood culture positive isolates. Whereas this may reflect the relative incidence of organisms in a Ghanaian tertiary care setting biased by its referral policies, we were unable to determine if these were nosocomial incidents or community acquired infections because of insufficient data on inpatient and outpatient status. Another issue worth mentioning is the unavailability of data on nalidixic acid screening for the detection of reduced susceptibility of fluoroquinolone. To test for *in vivo* fluoroquinolone resistance, *in vitro* nalidixic acid is more appropriate. Quinolone-therapy will fail in spite of apparent ciprofloxacin sensitivity if first resistance mutations have occurred leading to *in-vitro* nalidixic acid resistance. Note also that by being retrospective, some patients had been stratified with predetermined definitions to which we were unable to fully assess clinical history for correlations that might contribute to the risk of TS or NTS infections. Patient clinical outcome data would have been very helpful in the contextual interpretation of the multi-drug resistant strain infections. Despite these shortcomings, our findings offer baseline information needed to create the awareness of salmonellae blood stream infections in Ghanaian hospitals, and also the need for surveillance and control.

## Conclusions

In conclusion, we report a 6.5% prevalence of Salmonella bacteraemia at the Korle-Bu Teaching Hospital; with a preponderance of NTS over TS. Children under five bear the brunt of the disease. Resistance to ciprofloxacin and cefotaxime, the recommended antimicrobial agents for treatment remains low; although multi drug resistance to other agents is high. In Ghana where Salmonella infections are endemic, it is important to maintain surveillance of the disease to allow for appropriate and timely interventions when required.

## Authors’ original submitted files for images

Below are the links to the authors’ original submitted files for images. Authors’ original file for figure 1 Authors’ original file for figure 2

## Abbreviations

μg: Microgram
ANOVA: Analysis of variance
ATCC: American Type Culture Collection
BEMP: Bone infections, endocarditis, meningitis, pneumonia
BSI: Blood stream infections
CI: Confidence interval
CLSI: Clinical and Laboratory Standards Institute
r^2^ or r_s_^2^: Coeeficient of determination
Crx res.: Cefuroxime resistance
ESD: Eric Sampene-Donkor
Jan-Mar: January to March
KBTH: Korle-Bu Teaching Hospital
LAK: Labi Appiah-Korang
LOWESS: locally weighted scatter plot smoothing
MDR: Multidrug resistance
NICU: Neonatal intensive care unit
NJ: New Jersey
NOA: Naa Okaikor Addison
NON: Noah Obeng-Nkrumah
NTS: Non-typhoidal Salmonella
Oct-Dec: October to December
OR: Odds ratio
r: Pearson product–moment correlation coefficient
PMI: Princess-Marie Louis Children hospital
PUO: Pyrexia of unknown origin
ROC: Receiver Operating Characteristic
SD: Standard deviation
Sept: September
r_s_: Spearman’s rank correlation cooefficient
TS: Typhoidal Salmonella
USA: United States of America
