# Erratum: Salmonella blood stream infections in a tertiary care setting in Ghana

**DOI:** 10.1186/s12879-015-0947-3

**Published:** 2015-07-10

**Authors:** Appiah-Korang Labi, Noah Obeng-Nkrumah, Naa Addison, Eric S. Donkor

**Affiliations:** Department of Microbiology, Korle-Bu Teaching Hospital, Accra, West Africa Ghana; Microbiology Department, University of Ghana Medical School, Accra, West Africa Ghana

Following publication of [1] it has come to our attention that there was an error in the last author’s name which should be Eric S. Donkor and not Eric Sampene-Donkor.

We would like to sincerely apologize for the error and any inconvenience caused.
