# Supplementary figures and images for: Salmonella blood stream infections in a tertiary care setting in Ghana
Source: BMC Infect Dis. 2014 Dec 21;14:3857. doi: 10.1186/s12879-014-0697-7 (PMC4297363; doi:10.1186/s12879-014-0697-7)

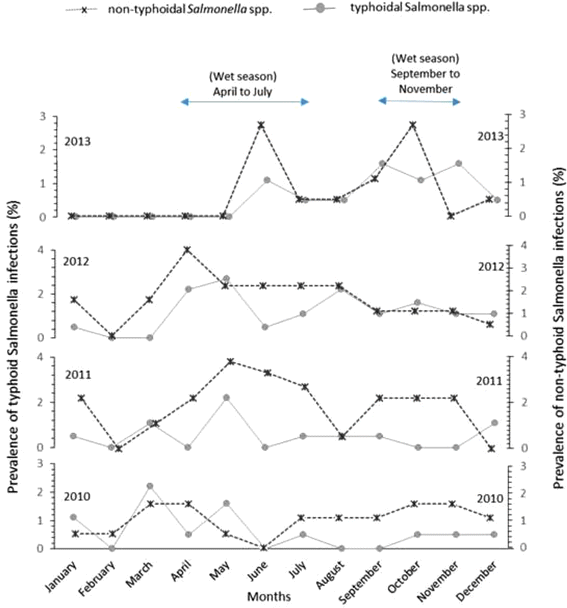

Supplement: Supplementary file 1 — Authors’ original file for figure 1 [file 12879_2014_697_MOESM1_ESM.gif]

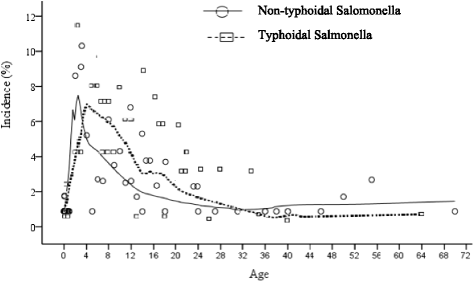

Supplement: Supplementary file 2 — Authors’ original file for figure 2 [file 12879_2014_697_MOESM2_ESM.gif]
